# *Desmodium intortum* (Mill.) Urb. Protein Isolate Aggregates as Pickering Stabilizers: Physicochemical Characteristics and Emulsifying Properties

**DOI:** 10.3390/molecules29163923

**Published:** 2024-08-20

**Authors:** Xuemei Tang, Hui Chang, Guanglong Yao, Jian Chen, Rongshu Dong

**Affiliations:** 1 Tropical Crops Genetic Resources Institute, Chinese Academy of Tropical Agricultural Sciences, Haikou 571101, China; tangxm1996@163.com; 2 Key Laboratory of Food Nutrition and Functional Food of Hainan Province, Engineering Research Center of Utilization of Tropical Polysaccharide Resources, Ministry of Education, School of Food Science and Engineering, Hainan University, Haikou 570228, China; changhui199907@163.com (H.C.); yaoguanglong@126.com (G.Y.); 3 National Key Laboratory for Tropical Crop Breeding, Sanya 572024, China

**Keywords:** protein resources, Pickering stabilizers, emulsion stability, high-value utilization

## Abstract

This work aimed to investigate the feasibility of fabricating Pickering emulsions stabilized by *Desmodium intortum* protein isolate (DIPI) aggregates. The DIPI aggregates were formed using heat treatment, and the effects of ionic strength and pH on their properties were investigated. The heat-treated protein exposes its hydrophobic groups due to structural damage, resulting in rapid aggregation of the protein into aggregates with a size of 236 nm. The results showed that the aggregates induced by ionic strength had larger particle size and higher surface hydrophobicity and partial wettability. Moreover, this study explored effective strategies for bolstering Pickering emulsion stability through optimized DIPI aggregate concentration (c) and oil fraction (ø). The DIPI Pickering emulsion (DIPIPE) formed at c = 5% and ø = 0.7 was still highly stable after 30 days of storage. As confirmed by laser confocal microscopy, DIPI aggregates could be adsorbed onto the oil–water interface to form a network structure that could trap oil droplets in the network. Collectively, the Pickering emulsion stabilized by DIPI aggregates exhibited excellent stability, which not only deeply utilizes the low-value protein resources in the *Desmodium intortum* for the first time, but also demonstrates the potential of DIPI for the bio-based field.

## 1. Introduction

Pickering emulsions refer to solid particle-stabilized emulsions, and their stabilization mechanism was first proposed by Ramsden [1] and S.U. Pickering [2]. The decisive advantage of the Pickering emulsion is that this system possesses significantly higher stability compared to conventional surfactant-stabilized emulsions due to the irreversible adsorption of solid particles at the interfaces of the oil–water interface [3], which has led to a wider application of Pickering emulsions in the food field [4,5]. In recent years, the use of biomolecules as Pickering stabilizers has become a hot research topic, and protein is one of the main sources of Pickering stabilizers. Many pioneering studies have reported protein-based Pickering stabilizers, such as whey protein [6,7], soy protein [8,9], and pea protein [10,11]. Lv [12] used Pickering emulsion prepared by whey protein isolate to encapsulate curcumin. The results showed that curcumin has the best stability against light degradation at the oil–water interface. Chen [13] exploited a ferritin-stabilized Pickering emulsion as a calcium phosphate biomimetic mineralization template and successfully obtained a hydroxyapatite capsule with a microcrystalline structure. The material had a promising perspective for the development of functional materials in the field of medicine. In recent years, the development of low-cost and effective novel protein Pickering stabilizers has gained significant attention.

After a simple heat treatment, the natural structure of the protein (including secondary, tertiary, and quaternary structures) is destroyed, exposing hydrophobic regions within the protein and forming aggregates [14,15]. The exposed hydrophobic region gives the protein aggregates better wettability and can more effectively reduce the surface tension at the water–oil interface, which contributes to better distribution of the protein at the interface and stabilization of the Pickering emulsion [16]. The aggregation of protein in solution is the result of intermolecular forces. The covalent cross-link, hydrophobic interactions, hydrogen bonds, and disulfide bonds can promote the aggregation of protein molecules, while electrostatic repulsion and steric hindrance can inhibit it. Therefore, ionic strength and pH values are important factors affecting protein aggregation. The increase in ionic strength of the solution can weaken the electric double layer around the protein molecules, creating electrostatic shielding and promoting protein aggregation [17]. Changes in pH values can alter the charge and amount of protein. Protein is neutral at its isoelectric point, the electrostatic repulsion is weak, and the interaction between molecules is significantly enhanced [18], which significantly promotes its aggregation.

Furthermore, Pickering stabilizers can be prepared by combining ionic strength or pH induction and thermal denaturation [19]. Liang [20] reported that pea protein isolate (PPI) can form particles with particle sizes ranging from 134 to 165 nm under acidic conditions with pH 3.0. Observed by atomic force microscopy, the pea protein isolate adopts the form of aggregate particles. Shen [21] reported that soy protein isolate dispersion undergoing treatments with heat followed by a certain concentration of NaCl can be aggregated into nanoparticles. This colloidal particle can be used to stabilize Pickering emulsions.

*Desmodium intortum* is a high-value crop with high crude protein content (up to 20%), which can serve as an emerging potential source of low-cost plant protein. The protein isolate extracted from this plant has not been applied to the research of Pickering emulsion. Therefore, it is interesting to evaluate the impact of *Desmodium intortum* protein isolate (DIPI) on the development and stability of Pickering emulsions. In the current work, a combination of heating and ionic strength adjustment was used to prepare DIPI aggregates, and the influence of DIPI aggregate concentration and oil fraction on the stability of Pickering emulsion was systematically investigated. The finding not only improves the utilization value of protein resources in *Desmodium intortum*, but also provides a new method for the development of protein-based Pickering stabilizers.

## 2. Results and Discussion

### 2.1. Characterization of DIPI Aggregates

**Figure 1:**
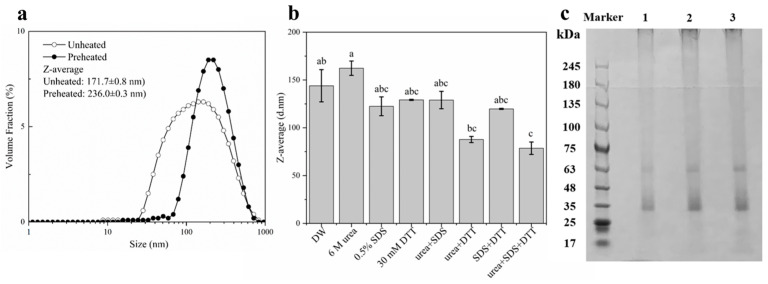
(**a**) The particle size distribution diagram of unheated and heated DIPI. (**b**) The influence of different protein perturbants on the average particle size of DIPI aggregates (significant differences are indicated by different lowercase letters (*p* < 0.05)). (**c**) SDS-PAGE patterns of DIPI aggregates (1: 0.5 mg/mL, 2: 1 mg/mL, 3: 1.5 mg/mL).

After heating at 95 °C for 30 min, DIPI aggregates were obtained and their average particle size was then determined. It should be noted that agglomerates are used for destroyed protein particles, and sometimes they can be used alternatively with particles. As can be seen in Figure 1a, compared with the unheated DIPI (171.7 ± 0.8 nm), the average particle size of aggregates increased to 236.0 ± 0.3 nm, the particle size distribution moved in the direction of larger particle sizes, and the size distribution became more distinct as the proportion between 20 and 80 nm disappeared. This shows that the heat treatment can lead to the aggregation of the DIPI. The aggregation of protein molecules may be due to the unfolding of peptide chains induced by the thermal energy, so the hydrophobic groups within the protein molecules were exposed while the number of hydrophilic groups was reduced [14]. At the same time, heat treatment can increase the probability of molecular collisions, which can lead to the aggregation of proteins [22]. Lv et al. [12] used heat treatment for the preparation of protein aggregates and demonstrated that they can be used as effective Pickering stabilizers.

In the presence of different protein-perturbing agents, the change in average particle size of DIPI aggregates can be used to determine the intermolecular forces between protein molecules in the aggregates. The dodecyl moiety of sodium dodecyl sulphate (SDS) consists of a hydrophobic structure that can be inserted into the hydrophobic interior of proteins, thereby disrupting their hydrophobic interactions. The structure of urea is highly similar to the peptide groups that comprise proteins. Urea has a stronger ability than water to form hydrogen bonds with proteins and can also destroy the hydrogen bonds within protein molecules [23]. Dithiothreitol (DTT) can prevent the formation of intramolecular or intermolecular disulfide bonds between cysteines in proteins [24]. The protein-perturbing agents used in our experiment were 6 M urea, 0.5% SDS, 30 mM DTT, or various mixtures of these. As shown in Figure 1b, the particle size was reduced in the presence of either 0.5% SDS alone or 30 mM DTT alone. Nevertheless, the effect of SDS was more pronounced than that of DTT. In the presence of a mixture of the two agents, the particle size also decreased, more significantly than in the presence of urea or DTT alone. In addition, the particle size decreased significantly when all three agents were combined together.

Therefore, it can be inferred that the forces maintaining the DIPI aggregates are hydrophobic interactions and disulfide bonds. Thermal aggregation of protein molecules is the result of various intermolecular forces [25]. The hydrophobic interactions and covalent cross-link through disulfide bonds can promote the aggregation of protein molecules. Furthermore, strong intermolecular disulfide bonds can improve the integrity of protein aggregates. The aggregates did not dissociate easily when placed at the oil–water interface of the emulsion.

SDS-PAGE analysis was performed to investigate the molecular weight distribution of DIPI aggregates. As presented in Figure 1c (lanes 1, 2, and 3), two major protein bands were observed at approximately 35 and 63 kDa, and it can be speculated that these two fractions may have been the main components of DIPI aggregates.

### 2.2. Influence of Ionic Strength and pH on the Characteristics of DIPI Aggregates

#### 2.2.1. Size, Zeta Potential, and Surface Hydrophobicity: Modulation of Ionic Strength

**Figure 2:**
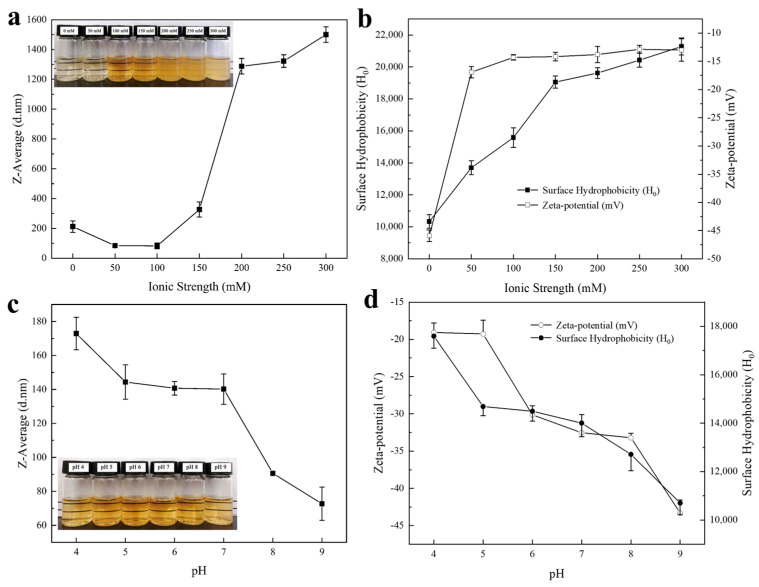
(**a**) The influence of ionic strength on the average particle size (inset: the appearance of DIPI aggregates at different ionic strengths). (**b**) The influence of ionic strength on the zeta potential and H_0_. (**c**) The influence of pH on the average particle size (inset: the appearance of DIPI aggregates under different pH). (**d**) The influence of pH on the zeta potential and H_0_.

As can be seen in Figure 2a, the average particle size of DIPI aggregates (1%, *w*/*v*) at an ionic strength of 50 mM and 100 mM was significantly smaller than that without salt ions. As the ionic strength was increased from 100 mM to 150 mM, the average particle size of the aggregates increased significantly from 83 nm to 327 nm. When the ionic strength was further increased to 300 mM, the average particle size further increased and reached the micron level of 1.5 μm. This is consistent with the observations shown in the inset. At low ionic strengths (0–150 mM), the particle size was small and the dispersion remained clear. At high ionic strengths (200–300 mM), the particle size increased and the dispersion became turbid. The decrease in average particle size of DIPI aggregates was attributed to the dissolution of protein under low-ionic-strength conditions, while the increase in average particle size may have been mainly caused by the competition between protein and salt ions to bind water molecules for solvation. In addition, high salt concentration can induce the binding between water molecules and salt ions, weakening the interaction between protein and water molecules. According to the research of Wen et al. [26], it was also found that the particle size of protein aggregates increased with the increase in ionic strength.

As can be seen in Figure 2b, in the absence of salt ions, the DIPI aggregate had a relatively high negative charge (−46 mV) and was dispersed in the aqueous solution due to strong electrostatic repulsion. Upon the addition of salt ions (50–300 mM), an electrostatic shielding effect was generated. When the ionic strength was 50 mM, the zeta potential value was −17 mV, indicating that the negative charge was largely shielded. When the ionic strength was increased to 300 mM, the zeta potential of the DIPI aggregates decreased to −13 mV. It is worth noting that the surface of the protein aggregates was always negatively charged (it was never converted to a positive charge), indicating that the particles can tolerate salt ions to some extent. Moreover, the addition of salt ions also increased the surface hydrophobicity (H_0_) of protein (Figure 2b) and reduce the intermolecular repulsion, causing the protein molecules to aggregate easily. With the increase in ionic strength, H_0_ increased from 10,338 to 21,277. It is generally believed that increasing ionic strength can cause hydrophobic groups to become more buried [27]. This study is different from the traditional concept, which may be due to the increase in ionic strength being able to reduce the negative charge on the protein surface, thereby weakening the electrostatic repulsion between ANS^−^ and protein. This allows more ANS^−^ and cationic groups of protein molecules to form ionic pairs [28].

#### 2.2.2. Size, Zeta Potential, and Surface Hydrophobicity: Modulation of pH

As displayed in Figure 2c, as the pH increased from 4 to 9, the average particle size of DIPI aggregates (1%, *w*/*v*) decreased from 173 nm to 73 nm, and the absolute zeta potential also increased from 19 mV to 43 mV. The isoelectric point (pI) of DIPI was determined to be 2.0. When the pH is close to pI, the interactions between protein molecules and water molecules are reduced, the net charge is also lower, and aggregates with larger particle sizes can be formed. When the pH is greater than pI, the protein is negatively charged [18], and the interactions between water molecules and negatively charged molecules can lead to solubilization. The more the pH deviates from pI, the more charge the protein carries, resulting in a higher potential value that makes it easier to generate electrostatic repulsion between protein molecules. Theoretically, stronger electrostatic repulsion between particles can prevent the aggregation of particles in solution, resulting in high dispersion [29]. Based on the appearance of DIPI aggregates at different pH values (inset), it can be observed that the dispersion was transparent at pH values of 4–9, which is consistent with the result for the change trend of zeta potential. In addition, the surface hydrophobicity decreased steadily with increasing pH (Figure 2d). This is probably because the higher the solubility of protein, the fewer hydrophobic groups on its surface.

#### 2.2.3. Contact Angle and SEM

Ionic strength and pH can affect the interfacial wettability of DIPI aggregates, thus determining their distribution and adsorption capacity at the interface, which is the key to determining whether solid particles can be used as Pickering stabilizers [29].

**Figure 3:**
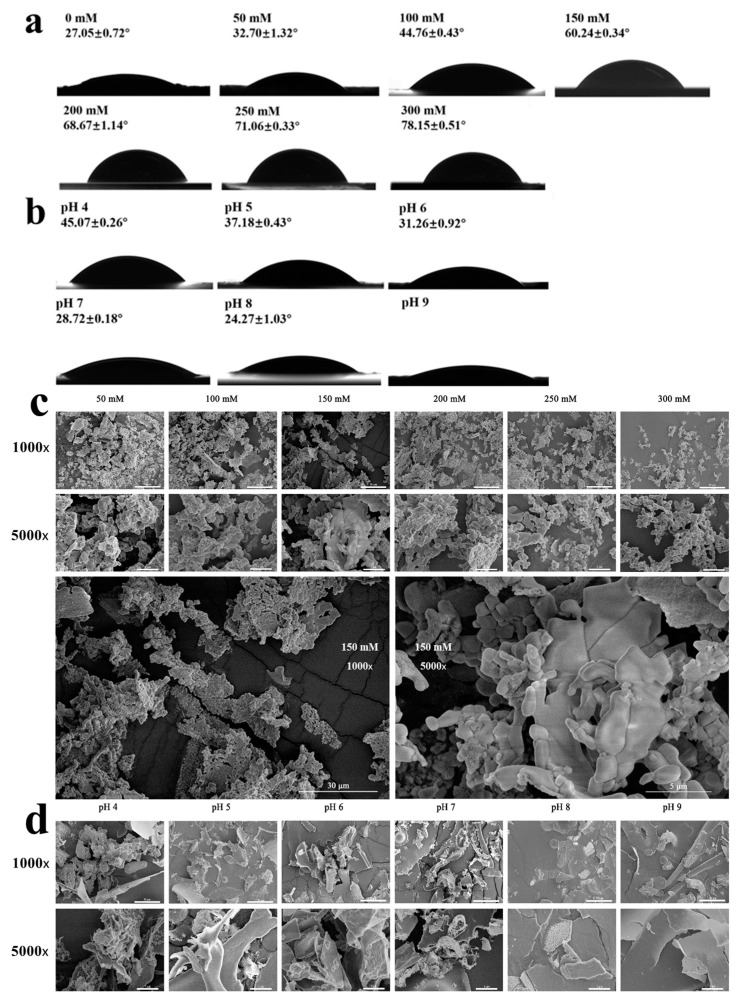
(**a**,**b**) Contact angles under different ionic strength and pH. (**c**,**d**) SEM images under different ionic strength and pH. The bars for the 1000× and 5000× images are 30 μm and 5 μm, respectively.

Figure 3a,b showed the three-phase contact angles of aggregates at different ionic strengths and pH values. When the pH ranged from 4 to 9 and the ionic strength ranged from 0 to 100 mM, the contact angle of the aggregate particles was less than 45°, with good hydrophilicity. It is worth noting that DIPI aggregates had stronger hydrophilicity at a pH of 9 and had a smaller contact angle (the exact contact angle could not be calculated because the equation could not be fitted with the data). The contact angle decreased with increasing pH, which is consistent with previously reported results [30]. The reason for the decrease in the contact angle could be that the increase in pH exposed more hydrophilic groups of DIPI aggregates. In contrast, when the ionic strength was in the range of 100–300 mM, the contact angle of the particles was relatively large, closed to 90°, and had better two-phase wettability, which was suitable for preparing the oil-in-water (O/W) Pickering emulsions. This observation agreed with a previous study reported by Ning et al. [19]. The increase in contact angle with the increase in ionic strength was likely due to DIPI aggregates exposing more hydrophobic groups. The above results are consistent with the H_0_ determination. Higher ionic strength resulted in higher surface hydrophobicity and increased the contact angle, while higher pH resulted in lower surface hydrophobicity with a smaller contact angle. At an ionic strength of 200–300 mM, the particle size of the DIPI aggregates increased sharply and the particles were very unstable, causing them to aggregate and precipitate. Thus, the DIPI aggregate dispersion with an ionic strength of 150 mM was selected to prepare Pickering emulsions in a follow-up study.

To further verify the previous analysis, Figure 3c,d show the SEM micrographs of freeze-dried DIPI aggregate powder at different ionic strengths and pH values. As can be seen from Figure 3c, the increase in ionic strength led to an intensification of particle aggregation. DIPI aggregates tended to be aligned towards roughly spherical and rod-like shapes. When the ionic strength was more than 150 mM, the degree of particle aggregation increased significantly. Under different pH values, adjacent DIPI aggregates fused together and had lamellar structures. The increase in pH caused the surface charge of protein particles to increase significantly, which considerably increased the interactions between protein and water molecules.

### 2.3. Characteristics of Pickering Emulsions Stabilized by DIPI

#### 2.3.1. Influence of DIPI Concentration and Oil Fraction on Emulsion Rheological Properties

**Figure 4:**
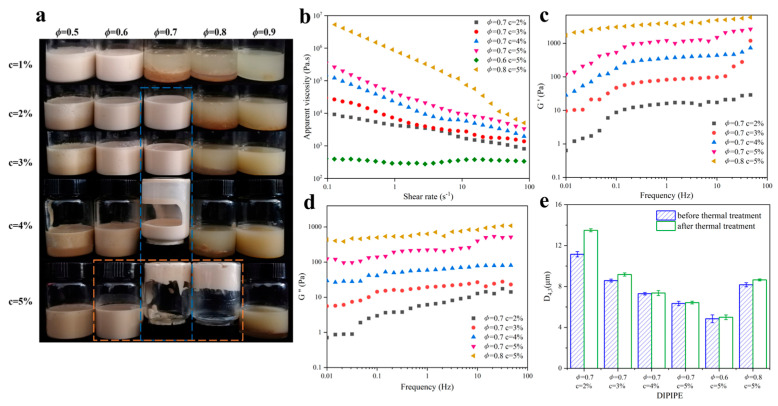
(**a**) The appearance of the emulsion after standing for 1 h. Rheological properties of emulsions prepared under different protein concentration (2–5%) and oil fraction (0.6–0.8); (**b**) the variation in apparent viscosity of emulsion with shear rate; (**c**,**d**) frequency dependence profiles of G′ and G″ of the Pickering emulsion. (**e**) Effect of thermal treatment on droplet size (D_4,3_) of the Pickering emulsion.

Figure 4a presented the appearance of unstored Pickering emulsions with different DIPI concentrations (c, 1–5%) and oil phases (ø, 0.5–0.9) after standing for 1 h. Pickering emulsion is divided into two types: water in oil (W/O) and O/W. To determine the type of emulsion, the emulsion is usually dropped into water or oil. The emulsion that can be evenly dispersed in water is considered an O/W-type emulsion, otherwise, it is a W/O-type emulsion. Furthermore, at a stabilizer contact angle of less than 90°, an O/W-type Pickering emulsion is preferably formed and, conversely, a W/O-type Pickering emulsion is formed. According to the test results, all the emulsions shown in Figure 4a were O/W emulsions. It can also be seen from the figure that at c = 1%, with the increase in the oil fraction, the number of particles adsorbed per unit area decreased, which was not sufficient to stabilize the emulsion. At ø = 0.5, as the particle concentration increased, the protein particles that were not adsorbed at the interface dissolved and flocculated, and the Pickering emulsion was not formed. Thus, the emulsions with ø = 0.7 and c = 2–5%, and those with c = 5% and ø = 0.6–0.8, were selected in subsequent experiments in which their properties were observed.

Figure 4 shows the rheological properties of the freshly prepared DIPI Pickering emulsion (DIPIPE) after standing for 1 h. As shown in Figure 4b, the viscosity of all emulsions except the emulsion with ø = 0.6 and c = 5% decreased with the increase in shear rate, indicating that shear thinning occurred in the emulsions, which was likely due to the presence of the flocculation structure in the emulsions. Notably, the shear viscosity increased with the increase in protein concentration and ø. From their appearances (Figure 4a), it can also be seen that the emulsions with a high protein concentration (5%) and a high oil ratio (0.7 or 0.8) had high viscosity, low fluidity, and could be inverted. The flocculation of droplets in the emulsions can lead to the formation of a gel network structure. It can also form a self-supporting paste emulsion under a high protein concentration or oil fraction.

Figure 4c,d showed the dependence of the storage modulus (G′) and loss modulus (G″) on the frequency of different DIPIPE samples. Since the emulsion (ø = 0.6, c = 5%) was flexible with low viscosity, its G′ and G″ values could not be measured within the performed frequency sweep range. For other emulsions, the G′ value was always higher than the G″ value in the measured frequency range. Cui also found G′ > G″ in Pickering gel samples [31]. This indicated that these emulsions have a gel-like structure and are mainly elastic in this frequency range. As the protein concentration and ø increased, the G′ value gradually increased, indicating that the gel network structure of the emulsion was strengthened. The gel strength of the emulsions with c = 5% and ø = 0.7 and 0.8 was significantly greater than that of other emulsions. This may be because more protein particles were adsorbed on the surface of the oil droplets, which continuously strengthened aggregation and cross-linking. The gel network structure can wrap and grab more dispersed oil droplets, thereby significantly improving the gel properties [32].

Figure 4e showed that the thermal treatment environment at 85 °C had only a slight effect on the droplet size change of the DIPIPE. The D_4,3_ of the emulsion (ø = 0.7, c = 2%) increased by 2.34 μm, and the D_4,3_ of the emulsion (ø = 0.7, c = 5%) increased only by about 0.086 μm. In summary, DIPIPE exhibited excellent thermal stability at 85 °C.

#### 2.3.2. Influence of DIPI Concentration on Emulsion Droplet Size and Microstructure

**Figure 5:**
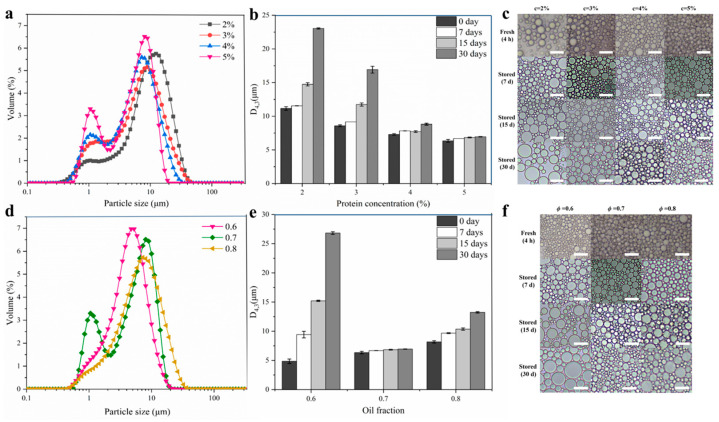
Influence of DIPI concentration on emulsion droplet size and microstructure (ø = 0.7, c values of 2–5%): (**a**) the particle size distribution of fresh Pickering emulsion; (**b**) changes in the D_4,3_ of the emulsion with storage time; (**c**) changes in the microstructure of the emulsion with storage time. Bars: 20 μm. Influence of oil fraction on emulsion droplet size and microstructure (c = 5%, ø values of 0.6–0.8): (**d**) the particle size distribution of fresh Pickering emulsion; (**e**) changes in the D_4,3_ of the emulsion with storage time; (**f**) changes in the microstructure of the emulsion with storage time. Bars: 20 μm.

Figure 5a,b depict the droplet size distribution of freshly prepared Pickering emulsion with different protein concentrations and the change in droplet size of Pickering emulsion during storage. The particle sizes of all Pickering emulsions were in the micron range. For the freshly prepared emulsion, the increase in protein concentration from 2% to 5% caused the main peak of the particle size distribution to shift to the direction in which small particle sizes were distributed, and D_4,3_ also decreased from 11.15 μm to 6.34 μm. A low protein concentration can result in fewer particles covering the surface of the oil droplets, leading to the formation of larger droplets. In contrast, when the protein concentration was increased, the size of the emulsion droplets gradually decreased. The reason for this may be that the particles that can cover the surface of the oil droplets increased, reducing the particle size and improving the stability of the emulsion. In Figure 5a, small peaks of around 1 μm could be seen, which may have been small emulsion droplets or DIPI aggregate particles. Throughout the storage period, the changes in D_4,3_ of the emulsion tended to increase, and when the storage time reached 15 and 30 days, the D_4,3_ of the emulsions with low protein concentrations (2% and 3%) was significantly increased. However, throughout the storage period, the changes in D_4,3_ of emulsions with high protein concentrations (4% and 5%) were small, whereas the changes for the emulsions with c = 5% were the smallest (increased from 6.34 μm to 6.95 μm). Han et al. also found that as the number of particles increased, the size of oil droplets decreased and stabilized [33].

The microstructures of the emulsion droplets were observed under an optical microscope (Figure 5c). The observations show that the structure of the Pickering emulsion droplets was complete. With the increase in protein concentration, the size of the droplets gradually decreased. When the storage time was extended from 0 to 30 days, an emulsion with a protein concentration of 5% showed very little change in droplet size and microstructure. These observations are consistent with the particle size date described above, indicating that DIPIPE prepared at c = 5% was a stable Pickering emulsion.

#### 2.3.3. Influence of Oil Fraction on Emulsion Droplet Size and Microstructure

Figure 5d,e show the droplet size distribution of freshly prepared Pickering emulsions with different oil fractions and the size change of Pickering emulsion droplets during storage. In contrast to the influence of protein concentration, with the increase in ø from 0.6 to 0.8, the main peak of the particle size distribution shifted toward large particle sizes and the D_4,3_ increased from 4.85 μm to 8.17 μm. At ø = 0.6 and c = 5%, the emulsion could be successfully formed (Figure 4a), showing that the concentration of nanoparticles at this time was sufficient to stabilize the current oil phase volume fraction. However, as the oil fraction increased, the droplet size became larger, which may have been caused by the decrease in the number of adsorbed protein particles per unit area. From Figure 5d, the emulsion with ø = 0.7 also had a small peak at around 1 μm, which may also have been small droplets or DIPI aggregate particles. In the storage experiment (Figure 5e), the extension of the storage time caused the droplet size of the emulsion to increase sharply with ø = 0.6, which may have been due to poor viscoelasticity of the emulsion with ø = 0.6. On the contrary, when ø was 0.7 or 0.8, the emulsion had a three-dimensional network structure, and the oil droplets were wrapped in the grid structure. However, a continuous increase in the oil fraction caused a decrease in the stability of the emulsion. The microstructure of the emulsion shown in Figure 5f indicates that as ø gradually increased, the size of the droplets increased, and during storage, when ø was 0.6 or 0.8, the droplet size and the microstructure of the emulsion changed significantly. These observations are also consistent with the particle size results and show that the emulsion prepared at ø = 0.7 had excellent stability.

#### 2.3.4. Influence of DIPI Concentration and Oil Fraction on Emulsion Creaming Index

**Figure 6:**
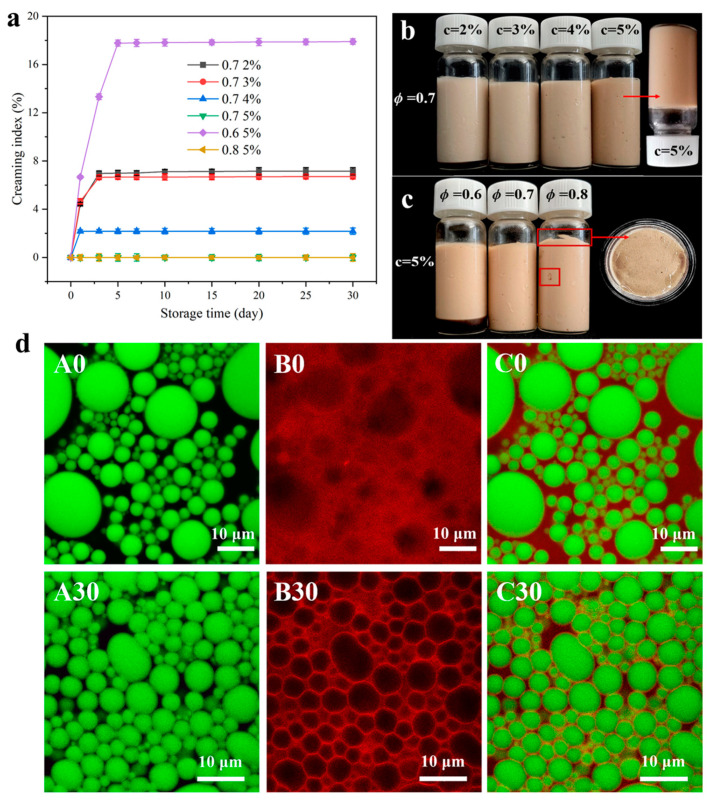
(**a**) Changes in the creaming index of different Pickering emulsions during storage. (**b**,**c**) Appearances of emulsions with different protein concentrations and oil fractions after 30 days of storage. (**d**) CLSM image of DIPI-stabilized Pickering emulsion (c = 5%, ø = 0.7).

In this experiment, the changes in creaming index (CI) of all emulsions stored at 4 °C for 30 days were measured (Figure 6a). According to the results, CI gradually decreased with the increase in protein concentration. Additionally, as the oil phase increased, the rate of emulsification decreased significantly. Comparing all emulsions, the emulsification index of the emulsion with c = 2–4% and ø = 0.7 and that with ø = 0.6 and c = 5% increased rapidly in the first 3 days of storage, and the rate of increase slowed down gradually until it stabilized after 5 days. In contrast, the CI% of the emulsion with c = 5% and ø = 0.7 and 0.8 remained at zero during the entire storage process, suggesting that the emulsion was stable. This may be due to the fact that higher protein concentration and oil fraction were more conducive to the formation of the gel network structure and increased the viscosity of the system (Figure 4), thereby inhibiting the emulsification to a certain extent. The above results could also be confirmed by the appearance of the emulsion after 30 days of storage (Figure 6b,c). The stratification of the emulsion was caused by the difference between the density of the dispersed phase and the continuous phase, forming a separated phase characterized by the appearance of the dispersed phase at the bottom of the emulsion. At c = 2–3% (ø = 0.7) and ø = 0.6 (c = 5%), a clear precipitation of the water phase was observed at the bottom of the emulsion. There were also oil leaks on the surface and inside the emulsion with ø = 0.8 (c = 5%). A comprehensive analysis of the results proves that DIPIPE prepared under c = 5% and ø = 0.7 could stabilize the emulsion most effectively and could still be inverted even after 30 days of storage.

#### 2.3.5. Confocal Laser Scanning Microscopy Analysis of DIPIPE

Figure 6d displays the confocal laser scanning microscope images of freshly prepared DIPIPE (c = 5%, ø = 0.7) stored for 30 days. The green color represents the oil phase structure, and the red color represents the protein structure. An overlay of the images (Figure 6(dC0,C30)) shows that the oil droplets were wrapped in the protein to form a stable O/W-type Pickering emulsion. For the freshly prepared emulsion, many bright red spots were observed in the water phase (Figure 6(dB0)), indicating that many protein aggregates were not completely adsorbed at the interface. However, it was found that the DIPI aggregates were adsorbed at the oil–water interface, forming an interface layer and a red grid structure, which could prevent the merging and coalescence of the droplets (Figure 6(dB30)). Because the oil droplets were wrapped around by protein, causing them to be closely connected or squeezed, the fluidity of the emulsion was limited, and as a result, a stable Pickering emulsion was formed. This experimental phenomenon is similar to the findings of Gu et al. [34].

## 3. Materials and Methods

### 3.1. Materials

*Desmodium intortum* (Mill.) Urb. was collected from Jianfengling in the southwest of Hainan Province. Soybean oil was purchased from a local supermarket (Haikou, China) without further purification. Sodium hydroxide (NaOH), hydrochloric acid (HCl), sodium chloride (NaCl), and hydrogen peroxide (H_2_O_2_) were purchased from Sinopharm Chemical Reagent Co., Ltd. (Beijing, China). All reagents were of analytical grade.

### 3.2. Preparation of DIPI Aggregates

DIPI was prepared by alkali extraction and acid precipitation methods. Desmodium intortum were dried at 60 °C, crushed and sieved with a 40-mesh sieve, degreased with petroleum ether, and then naturally dried. Defatted powder was mixed with deionized water at a ratio of 1:37 (*w*/*v*), and the pH of the solution was adjusted to 10 with 1.0 M NaOH. The mixture was stirred continuously for 1.5 h, during which time the pH was adjusted to 10 every 15 min. The mixture was then separated, and the supernatant was collected after passing through a 100-mesh sieve and then decolorized with 30% H_2_O_2_ at 40 °C for 1 h. The pH of the supernatant was adjusted to 2.0 (the isoelectric point of the DIPI) to precipitate the protein. The precipitate was washed several times with deionized water. After the pH was adjusted to 7, the precipitate was stirred until completely dissolved. After that, it was dialyzed against deionized water before being subjected to lyophilization. The DIPI obtained was not the pure protein of a single type, but a mixture containing many proteins. The protein content in DIPI (total nitrogen × 6.25) was 90.7% ± 0.46%, determined using the Kjeldahl method (wet basis).

The preparation of DIPI aggregates was based on the method of Liu, with some modifications [20]. A certain mass of freeze-dried DIPI sample was accurately weighed and then dispersed in deionized water (5%, *w*/*v*). After stirring for 2 h at room temperature, the solution was stored at 4 °C overnight to allow the proteins to be fully hydrated. The solution was then centrifuged to remove traces of insoluble substances. The DIPI solution was heated at 95 °C for 30 min, and then immediately cooled in an ice bath to generate DIPI aggregates. Next, the DIPI aggregate solution was diluted to 1% and then divided into several parts to study the effects of ionic strength (0–300 mM) and pH (4–9) on the properties of DIPI aggregates. After maintaining the pH at 7, NaCl was added to the aggregate dispersion to prepare DIPI aggregate solutions with different ionic strengths, while the pH of the aggregate dispersion was adjusted to different values without adding NaCl. All DIPI aggregate dispersions were allowed to stand and stabilize for 2 h before being subjected to characterization.

### 3.3. Characterization of DIPI Aggregate

#### 3.3.1. Size Distribution, Particle Size, and Zeta Potential

The particle size of DIPI aggregates was determined by dynamic light scattering (DLS) using a Zetasizer Nano ZS90 (Malvern Instruments, Malvern, UK) particle size analyzer, which is more suitable for nanoscale particle size analysis. To avoid the multiple-particle effects, the sample was diluted 100 times with deionized water while maintaining its ionic strength or pH value. The test temperature was set at 25 °C. The diluted sample was transferred to the sample cell (model DTS 1060C) and then subjected to the same instrument for measurement of the zeta potential.

To investigate the cohesive interactions within DIPI aggregates, the particle size changes in the aggregates dispersed in different solvents were measured. DIPI aggregates were dispersed in distilled water, 6 M urea, 0.5% SDS, 30 mM DTT, and mixtures of these for 30 min, and then the particle size of the aggregates was measured. The data presented are the average of three measurements.

#### 3.3.2. Surface Hydrophobicity (H_0_)

H_0_ was determined using a fluorescent probe ANS. Four mL of PBS (10 mM, pH 7.0) were added to five test tubes, and then 10, 20, 30, 40, and 50 μL of 1% protein aggregate dispersion were added and mixed in each case. Twenty μL of ANS^−^ solution (8.0 mM) were quickly added to the above mixture. The F7000 fluorescence spectrophotometer (Hitachi Co., Tokyo, Japan) was employed to measure the fluorescence intensity at an excitation wavelength of 370 nm and an emission wavelength of 470 nm. The fluorescence intensity was plotted as the ordinate and the protein concentration as the abscissa, and H_0_ was calculated as the slope of fluorescence intensity as a function of the protein concentration (g/mL).

#### 3.3.3. Contact Angle Measurements

The contact angle of the protein aggregates was measured with an optical surface analyzer (OSA100C, Ningbo NB Scientific Instruments Co., Ningbo, China). The prepared aggregates were freeze-dried and pressed into thin slices before being placed on a microscope slide. A drop of 2 μL of ultrapure water was dropped onto the thin slice using a high-precision syringe, and the contact angle was determined by fitting the data with the Laplace–Young equation. The data were presented as an average of 10 measurements.

#### 3.3.4. Scanning Electron Microscopy (SEM)

The morphologies of the protein aggregates were observed using a Thermo Verios G4 UC scanning electron microscope (Thermo Fisher Scientific Co., Waltham, MA, USA). The protein powder was secured on an aluminum stage with a conductive tape and then sputter-coated with gold. The observation was carried out at an acceleration voltage of 10 kV, and SEM images (1000× and 5000× magnifications) were obtained.

### 3.4. Characterization of DIPIPE

#### 3.4.1. Preparation of DIPIPE

The DIPI aggregate dispersion (5%, *w*/*v*) was prepared using the heating method described previously. While maintaining the pH of the dispersion at 7.0, NaCl was added to adjust the ionic strength (150 mM), and deionized water with the same pH and ionic strength was used to dilute the dispersion to different concentrations (c, 1–5%). To study the effect of protein concentration on the properties of the emulsion, the volume fraction of the oil phase was fixed (ø = 0.7), and to study the effect of the volume fraction of the oil phase (ø, 0.6–0.8) on the properties of the emulsion, the protein concentration was fixed (c = 5%). The protein water phase and the oil phase were mixed using an IKA-ULTRATURRAX T25 digital disperser (IKA 190 Works, Inc., Wilmington, NC, USA) at a speed of 20,000 rpm for 2 min to prepare the Pickering emulsion, with a total volume of 10 mL. The obtained emulsions were either subjected to analysis directly or stored at 4 °C in the emulsion stability experiments.

#### 3.4.2. Rheological Properties

A Hakke Mars rheometer (Typ006–1385, Thermo Fisher Scientific Co., Dreieich, Germany) was used to analyze the rheological behavior of the freshly prepared emulsion after standing for 2 h. The sample was placed between parallel plates (40 mm diameter) with a fixed distance of 1 mm, and the measurement temperature was 25 °C. The apparent viscosity experiment was measured at a frequency of 1.0 Hz and a shear rate in the range of 0.1–100 s^−1^. The frequency sweep experiment was measured at a strain of 0.2% (within the linear viscoelastic region) and a frequency range of 0.01–50 Hz, from which the relationship of the storage modulus (G′) and loss modulus (G″) with the frequency was obtained.

#### 3.4.3. Droplet Size Distribution and Volume-Averaged Droplet Size (D_4,3_)

The particle size of fresh or stored emulsion was measured with a BT-9300H (Bettersize Instruments Ltd., Dandong, China) laser particle size distribution analyzer, which is suitable for testing micrometer samples. The refractive index of soybean oil is 1.456 and that of water is 1.333. The emulsion was carefully dropped into deionized water, and the measurement was started when the obscuration of the intensity signal reached about 10%. The particle size of the emulsion was presented as D_4,3_. All samples were measured in parallel, and each measurement was repeated three times.

#### 3.4.4. Microstructure

The microstructure of the fresh or stored emulsion was observed using a biological microscope (BA210-T, Motic, Hong Kong, China) equipped with the Motic Image Advanced 3.2 system. A small amount of emulsion was dropped on a microscope slide and then covered with a glass slide before being observed under a microscope using a 40× lens, from which microscopic images of the emulsion were obtained.

#### 3.4.5. Creaming Index (CI)

The coalescence stability of the emulsion was evaluated by monitoring the layer formation of the emulsion after storage at 4 °C for different days and using the CI%. Two milliliters of fresh emulsion were added to a flat-bottomed glass bottle, and the bottle was then sealed with a sealing film to prevent the evaporation of water. Height (Hs) and total height (Ht) of the supernatant were recorded after storage of the emulsion, and whether oil leaked from the emulsion was also observed. The CI% was calculated as follows: CI% = (Hs/Ht) × 100%.

#### 3.4.6. Confocal Laser Scanning Microscopy (CLSM)

A confocal laser scanning microscope (TCS SP8, Leica Microsystems Inc, Wetzlar, Germany) was employed to observe the interfacial structure of the DIPI aggregate-stabilized Pickering emulsion. The sample was prepared according to the method of Dai and Dong [35,36]. The fluorescent dye Nile Red (0.1%, *w*/*v*) and Nile Blue (0.1%, *w*/*v*) were prepared with isopropanol. One milliliter of the emulsion was stained with 50 μL of Nile Red and 50 μL of Nile Blue. It was then dropped onto a glass slide and covered with a glass slide before observation. The excitation wavelength of Nile Red was 488 nm and the emission wavelength was 570 nm. After staining with Nile Red, the color of soybean oil became green. The excitation wavelength of Nile Blue was 633 nm and the emission wavelength was 672 nm. DIPI stained with Nile Blue dye was red in color.

## 4. Conclusions

A new type of Pickering stabilizer called DIPI aggregate was developed by sequential treatment with heat and by adjusting ionic strengths (150 mM). Different pH environments and ionic strength brought diversified effects on the properties of DIPI aggregates. In this study, DIPI aggregates adjusted by ionic strength were found to have better Pickering stabilizer properties, such as appropriate particle size and partial wettability. The Pickering emulsion stabilized by DIPI aggregates had similar properties to the previously reported emulsions stabilized by other particle types. Increasing the protein concentration was found to successfully generate an emulsion with smaller droplet size and higher anti-coalescing ability. Increasing ø could cause the droplet size to become larger, and a gel-like emulsion with better stability could be formed under a suitable ø (0.7). Finally, the CLSM micrographs may reflect the mechanism by which DIPI aggregates stabilize the Pickering emulsion. DIPI aggregates produced an interfacial layer around the oil droplets that prevented the aggregation of the oil droplets and stabilized the emulsions. This research not only exploits the low-value protein resources in the *Desmodium intortum*, but also provides a theoretical basis for the application of Pickering emulsion in industry. In future work, we intend to evaluate the ability of DIPIPE in delivering bioactive molecules or improving the function of food-grade biopolymers.

## Data Availability

Data will be made available on request.
